# Health Information Seeking Partially Mediated the Association between Socioeconomic Status and Self-Rated Health among Hong Kong Chinese

**DOI:** 10.1371/journal.pone.0082720

**Published:** 2013-12-13

**Authors:** Man Ping Wang, Xin Wang, Tai Hing Lam, Kasisomayajula Viswanath, Sophia S. Chan

**Affiliations:** 1 School of Public Health, The University of Hong Kong, Hong Kong SAR, China; 2 Center for Community-Based Research, Dana-Farber Cancer Institute/Department of Social and Behavioral Sciences, Harvard School of Public Health, Boston, Massachusetts, United States of America; 3 School of Nursing, The University of Hong Kong, Hong Kong SAR, China; Univ of Toledo, United States of America

## Abstract

**Background:**

Poor self-rated health (SRH) is socially patterned with health communication inequalities, arguably, serving as one mechanisms. This study investigated the effects of health information seeking on SRH, and its mediation effects on disparities in SRH.

**Methods:**

We conducted probability-based telephone surveys administered over telephone in 2009, 2010/11 and 2012 to monitor health information use among 4553 Chinese adults in Hong Kong. Frequency of information seeking from television, radio, newspapers/magazines and Internet was dichotomised as <1 time/month and ≥1 time/month. Adjusted odds ratios (aOR) for poor SRH were calculated for health information seeking from different sources and socioeconomic status (education and income). Mediation effects of health information seeking on the association between SES and poor SRH was estimated.

**Results:**

Poor SRH was associated with lower socioeconomic status (P for trend <0.001), and less than monthly health information seeking from newspapers/magazines (aOR = 1.23, 95% CI 1.07–1.42) and Internet (aOR = 1.13, 95% CI 0.98–1.31). Increasing combined frequency of health information seeking from newspapers/magazines and Internet was linearly associated with better SRH (P for trend <0.01). Health information seeking from these two sources contributed 9.2% and 7.9% of the total mediation effects of education and household income on poor SRH, respectively.

**Conclusions:**

Poor SRH was associated with lower socioeconomic status, and infrequent health information seeking from newspapers/magazines and Internet among Hong Kong Chinese. Disparities in SRH may be partially mediated by health information seeking from newspapers/magazines and Internet.

## Introduction

Self-rated health (SRH), a simple measure of general health, is useful in predicting morbidity and mortality.[1], [2] Determinants of SRH include demographic factors, socioeconomic status (SES), health behaviours and health status.[3], [4] Although studies have reported the effects of health communication on knowledge, perceptions, social norms and health behaviours,[5] its influence on SRH is seldom studied. The importance of health information seeking, a core dimension of health communication, is increasingly recognized as an important dimension of health care where patient-provider interactions are shifting from more paternalistic models to patient-oriented and consumer-driven models.[6] Studies among cancer patients have shown that health information seeking was associated with better quality of life, self-care management, treatment compliance and coping strategies.[7] In contrast, health information avoidance among cancer survivors was linked to poor SRH.[8] It is uncertain whether health information seeking among the general public was associated with better SRH.

Disparities in SRH, a proxy of health inequalities,[9] was well documented in people with lower SES reporting poorer SRH.[10] Apart from numerous factors that contribute to health inequalities, the Structure Influence Model posits that health inequalities may be partly explained by health communication inequalities defined as inequality in accessing, seeking, processing and using health information.[11] Health communication inequalities were observed in Western and Japanese populations in which people with lower SES having less frequent health information seeking, less attention and lower level of trust on health information.[12], [13].

Reports on the effects of health communication inequalities on SRH are sparse and most evidence is based on populations in the United States. Among the elderly, health literacy was found to be a mediator for linking the associations of ethnicity and education with SRH and preventive services use.[14] Similarly, health information avoidance was found to mediate the association between SES and SRH among cancer survivors.[8] Given that health communication is culturally sensitive in terms of information sources, messages and channels,[15] applicability of Western findings to Chinese populations is uncertain.

We have found that having lower SES was associated with infrequent health information seeking from mass media and Internet in Hong Kong,[16] the most westernised and economically developed city in China. Mass media in Hong Kong is vibrant owing to the complete freedom of speech and the universal coverage of television and radio broadcasting.[17] Daily newspaper circulation ranks 3^rd^ in Asia and 14^th^ in the world (222 circulations per 1000 people).[18] In recent years, newspapers readership is increasing due to the surge in number of free newspaper and the circulation.[19] Moreover, advanced cyber-infrastructure and relatively low cost of Internet access in Hong Kong are clear advantages to health information seeking. We therefore aim to assess the association of health information seeking with SRH among Chinese general public, and the mediation effects of health information seeking on disparities in SRH.

## Methods

### Ethical Statement

Ethical approval was granted by Institutional Review Board (IRB) of the University of Hong Kong/Hospital Authority Hong Kong West Cluster. Verbal informed consents were obtained and recoded verbatim, and the procedure was approved by the IRB.

### Survey design

As a part of the FAMILY Project (www.family.org.hk), the Hong Kong Family and Health Information Trends Survey (FHinTs) was conducted in 2009 (Nov-Dec), 2010/11 (Dec-Mar) and 2012 (Aug-Oct) using probability-based telephone surveys of the general public to monitor the opinions and behaviours on family health, information use and health communication. Detail survey design was reported elsewhere.[16], [20] In brief, Cantonese-speaking adults aged 18+ were interviewed through a two stage random sampling method with telephone numbers (seed numbers) were retrieved from residential telephone directories which covered about 76% of Hong Kong residents.[21] To capture the unlisted telephone numbers, new random telephone numbers were generated by plus or minus one or two of the last digit of the seed numbers. The telephone numbers were then listed in random order using a computer programme. Invalid household numbers, non-response calls and ineligible households were excluded. In the second stage, after interviewers introduced the study purpose, the adult respondent was asked how many eligible persons were living in the households. All eligible persons were listed and the one with the date of next birthday closest to the interview dates was selected. Each interview took about 20 minutes to complete. Among 6222 adults with confirmed eligibility, 4553 were successfully interviewed yielding a response rate of 73.2%. Sex and age distributions of the survey subjects were similar to Hong Kong census 2011 population data (Cohen’s effect sizes were small: 0.02 and 0.17) suggesting that the sample was quite similar to the general public.[22]

### Measurement

SRH was measured by asking the respondents “What do you think about your general health?” with responses of “excellent”, “very good”, “good”, “fair” and “bad”. Responses of “fair” or “bad” were categorised as poor SRH.[1] Frequency of health information seeking was assessed by 4 separate questions: “In the past 12 months, how often have you watched television for health related information?”. Similar questions were repeated to assess the frequency of health information seeking from radio, newspapers/magazines and Internet. Responses included “≥1 time/week”, 1–3 times/month”, “1 time in several months”, “rarely”, “no” and “never”. We dichotomised frequency of each source as <1 time/month and ≥1 time/month (reference) to distinguish frequent use as we were concerned with the skew distribution of health information as a continuous variable. Compared with the universal coverage of television and radio, health information seeking from newspapers/magazines and Internet were more likely to be associated with higher SES as shown in our previous study.[16] To measure the combined effects of newspapers/magazines and Internet, we combined the frequency of health information from newspapers/magazines and Internet and categorised it as “both ≥1 time/month” (reference), “either ≥1 time/month” and “both <1 time/month”.

Socioeconomic status (SES) was measured using educational attainment and household monthly income. Employment status was not included as our previous study found inconsistent associations between employment status and health information seeking.[16] Educational attainment was categorised as primary or below, secondary and tertiary or above. Monthly household income (HKD, 1 USD  =  7.8 HKD) was categorised as ≤$9,999, 10000–19999, 20000–29999, 30000–39999 and ≥40000. Doctor-diagnosed chronic diseases were recorded and dichotomised as none and any.

### Statistical analysis

STATA 10 was used for data analysis. All data were weighted by sex and age from Hong Kong 2011 census data. Binary logistic regression was used to yield adjusted odds ratios (aOR) of dichotomised SRH in relation to SES and health information seeking adjusting for demographic characteristics. Analyses were repeated using ordered logistic regressions by treating SRH as an ordinal variable as in the original reposes. Sources of health information seeking were mutually adjusted as only weak correlations between different sources were observed (correlation coefficients ranged from 0.03 to 0.32). Mediation effects of combined frequency of health information seeking from newspapers/magazines and Internet on the association between SES and SRH was assessed using Sobel’s test with P<0.05 indicated significant mediation.[23]–[25] Bootstrapping with 500 replications was used to estimate the standard error and 95% CI of direct and indirect effects.

## Results

Table 1 shows that nearly half (49.2%) of the respondents reported poor SRH which is more prevalent among female, older people, and people with lower educational attainment, lower household income and having chronic disease (all P for χ^2^ <0.001). Health information seeking from television, newspapers/magazines and Internet, but not radio, was significantly associated with lower likelihood of reporting poor SRH.

**Table 1:** Basic characteristics of subjects by poor self-rated health (SRH).

		All (Colum %)	All (Colum %)†	Poor SRH (49.2%)	
				Row %	p for χ^2^
Sex	Male	37.6	45.9	44.4	<0.001
	Female	62.4	54.1	53.4	
Age	18–24	12.6	10.5	40.2	<0.001
	25–44	23.9	37.6	45.4	
	45–64	47.1	36.4	52.7	
	≥65	16.4	15.6	56.0	
Education	≤Primary	18.7	14.8	62.2	<0.001
	Secondary	50.3	48.4	51.6	
	≥Tertiary	31.0	36.9	40.9	
Monthly household income	≤$9,999	22.4	19.4	59.7	<0.001
	$10,000–$19,999	24.5	23.5	54.7	
	$20,000–$29,999	20.7	21.4	45.9	
	$30,000–$39,999	14.3	14.2	42.7	
	≥$40,000	21.4	21.4	37.5	
History of chronic disease	Yes	64.3	67.8	67.5	<0.001
	No	35.7	32.2	40.5	
Health information seeking					
Television	Never/No	10.6	10.8	53.7	0.04
	<1 time/month	27.2	27.8	47.6	
	≥1 time/month	62.1	61.4	49.3	
Radio	Never/No	41.7	43.0	49.3	0.75
	<1 time/month	23.2	23.8	48.3	
	≥1 time/month	35.2	33.2	49.7	
Newspapers/magazines	Never/No	14.8	14.8	53.6	0.002
	<1 time/month	18.3	19.0	52.0	
	≥1 time/month	66.9	66.2	47.5	
Internet	Never/No	47.4	42.3	54.0	<0.001
	<1 time/month	20.3	22.1	48.6	
	≥1 time/month	32.3	35.6	44.0	

Weighted by sex and age of 2011 census data.

Being female, having chronic diseases, lower educational attainment and lower household income were associated with poor SRH (Table 2). Compared with monthly health information seeking from newspapers/magazines, less frequent health information seeking was associated with an aOR (95% CI) of 1.23 (1.07–1.42) for poor SRH (Table 3). The corresponding marginal non-significant aOR (95% CI) of1.13 (0.98–1.31) was observed for online health information seeking, and non-significant ORs for television and radio. Combined frequency of health information seeking from newspapers/magazines and Internet was linearly associated with higher odds of reporting poor SRH (P for trend <0.01). Similar results were observed using proportional odds ratios by treating SRH as an ordinal variable.

**Table 2:** Odds ratios (ORs) of poor SRH by socio-demographic characteristics.

	N	Adjusted ORs (95% CI) †	
		Binary logistic regression	Ordered logistic regression
Sex			
Male	2091	1	1
Female	2462	1.36 (1.20–1.54)***	1.35 (1.21–1.51)***
Age			
18–24	475	1	1
25–44	1704	1.30 (1.04–1.62)*	1.31 (1.08–1.58)**
45–64	1653	1.23 (0.98–1.55)	1.13 (0.92–1.37)
≥65	707	0.86 (0.65–1.13)	0.80 (0.63–1.01)
History of chronic disease			
No	3087	1	1
Yes	1466	2.89 (2.50–3.33)***	3.29 (2.88–3.75)***
Education			
≥Tertiary	1676	1	1
Secondary	2199	1.21 (1.05–1.41)**	1.12 (0.98–1.27)
≤Primary	671	1.51 (1.20–1.89)***	1.35 (1.10–1.65)**
P for trend	-	<0.001	<0.01
Monthly household income			
≥$40,000	848	1	1
$30,000–$39,999	565	1.21 (0.96–1.52)	1.16 (0.95–1.40)
$20,000–$29,999	851	1.36 (1.11–1.68)**	1.26 (1.05–1.50)*
≤19,999	1705	1.83 (1.50–2.22)***	1.61 (1.36–1.92)***
P for trend	-	<0.001	<0.001

Adjusting for year and mutually adjusted for variables in the table.

**P <0.01, ***P <0.001. P <0.05,

**Table 3:** Odds ratios of poor SRH by health information seeking.

Health information seeking	N	Adjusted ORs (95% CI) † ‡	
		Binary logistic regression	Ordered logistic regression
Frequency of individual sources †			
Television			
≥1 time/month	1747	1	1
<1 time/month	2783	0.96 (0.83–1.10)	0.91 (0.80–1.02)
Continuous scale§	-	1.00 (0.95–1.05)	0.98 (0.94–1.02)
Radio			
≥1 time/month	3035	1	1
<1 time/month	1511	1.03 (0.89–1.18)	1.06 (0.94–1.20)
Continuous scale§	-	1.01 (0.97–1.05)	1.02 (0.99–1.06)
Newspapers/magazines			
≥1 time/month	1534	1	1
<1 time/month	3008	1.23 (1.07–1.42)**	1.29 (1.14–1.46)***
Continuous scale§	-	1.05 (1.00–1.10)*	1.06 (1.02–1.11)**
Internet			
≥1 time/month	2930	1	1
<1 time/month	1621	1.13 (0.98–1.31)	1.10 (0.97–1.25)
Continuous scale§	-	1.02 (0.98–1.07)	1.02 (0.98–1.07)
Combined frequency ‡			
Both ≥1 time/month	1219	1	1
Either ≥1 time/month	2068	1.18 (1.01–1.39)*	1.20 (1.05–1.38)**
Both <1 time/month	1254	1.39 (1.16–1.68)***	1.42 (1.21–1.67)***
P for trend	-	<0.01	<0.001

Adjusting for sex, age, education, household income, chronic disease and years, and mutually adjusted for sources of health information seeking.

Combining frequency of health information seeking from newspapers/magazines and Internet. The aOR were adjusted for sex, age, education, household income, chronic disease, year, health information seeking from television and radio.

–3 times/month and ≥1 time/month as continuous scales. Treating the original frequency categories of never/no, seldom, 1 time/month, 1

**Figure 1:**
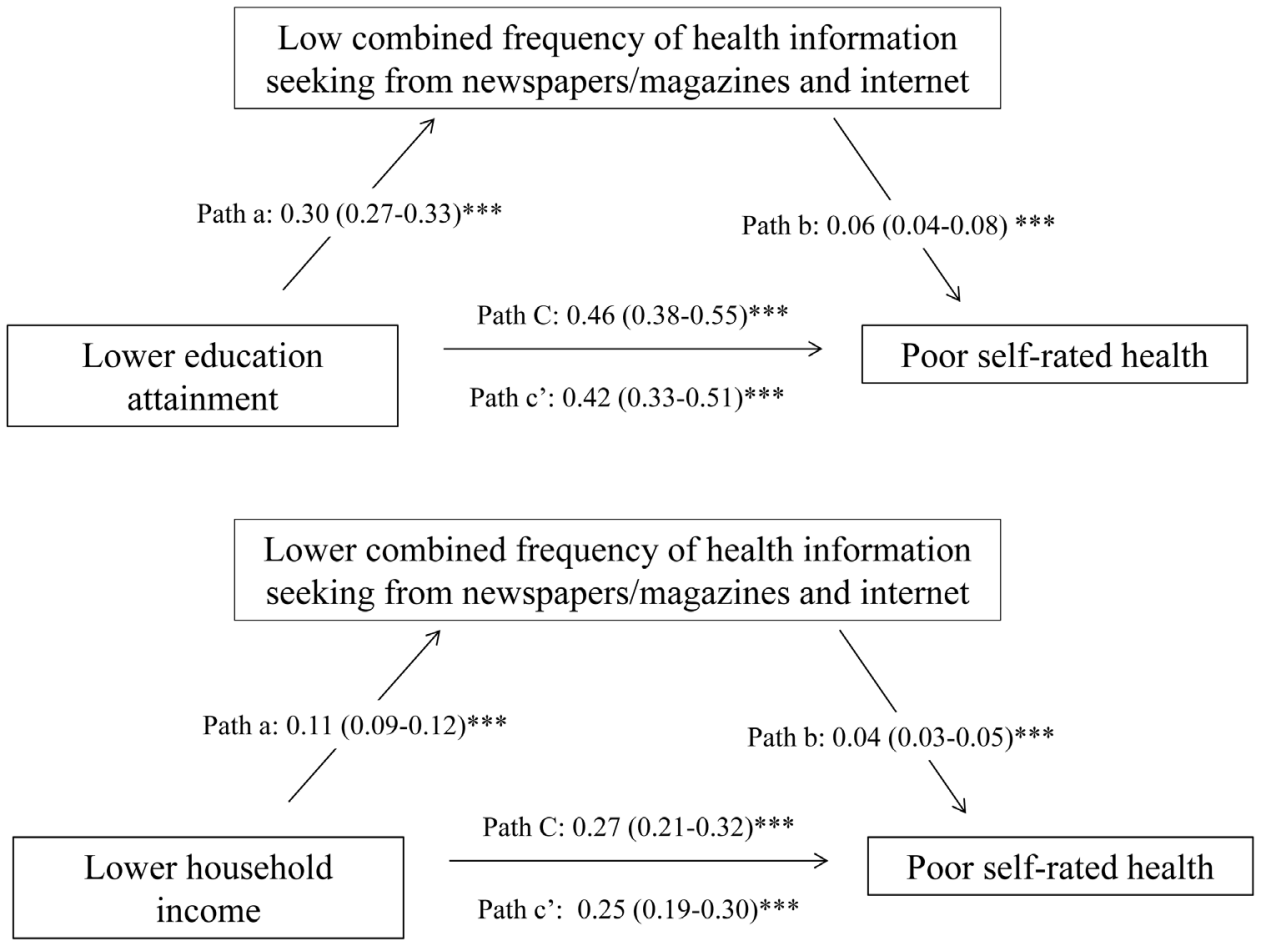
Mediation effects of information seeking from newspapers/magazines and Internet on disparities in poor SRH ^†^. ^†^ All figures are β-coefficients. ***P<0.001. Total effect of educational attainment on poor self-rated health was 17.4% (SE 0.017, 95% CI 14.1%–20.8%); indirect effect of information seeking on self-rated health was 1.6% (SE 0.5%, 95% CI 0.7%–2.6%), which yielded 9.2% of the total effect was mediated through health information seeking from newspapers/magazines and Internet (Sobel test P<0.01). Total effect of household income on poor self-rated health was 16.9% (SE 0.018, 95%CI: 13.7%–20.5%); indirect effect of information seeking on self-rated health was 1.3% (SE 0.3%, 95% CI 0.7%–2.0%), which yielded 7.9% of the total effect was mediated through health information seeking from newspapers/magazines and Internet (Sobel test P<0.01).

**P <0.01, ***P <0.001. P <0.05,

Figure 1 shows a slight decrease in β-coefficients for the association of educational attainment and household income with poor SRH after adjusting for health information seeking. Mediation analysis suggested that the associations were partially mediated (P for Sobel test <0.01) by health information seeking from newspapers/magazines and Internet. Educational attainment accounted for 17.4% of total effect on poor SRH, in which 9.2% of the total effects (17.4%×9.2%  = 1.6% points, 95% CI: 0.7%–2.6%) were mediated by health information seeking from newspapers/magazine and Internet. Similarly, health information seeking from newspapers/magazine and Internet accounted for 7.9% of 16.9% total effects (16.9%×7.9%  = 1.3% points, 95% CI 0.7%–2.0%) of household income on poor SRH.

## Discussion

We provided the first evidence that infrequent health information seeking from newspapers/magazines and Internet was associated with poor SRH among the general public in a non-Western population. Previous studies in the West among post-treatment cancer patients showed the beneficial effects of health information on SRH, probably through improving coping strategies, self-efficacy, decision making and social/cognitive functioning.[7], [8] Among the general public, health information seeking was found to strengthen health knowledge, awareness and self-efficacy, which may lead to positive behaviour changes.[26], [27] Studies have showed that frequent health information seeking was associated with healthy behaviours such as healthy diet, physical activity, cancer screening and less cigarette consumption.[28]–[30] Healthy lifestyles are protective for chronic diseases thus may result in better SRH.[31] Moreover, health information seeking was strongly associated with attention to the information which in turn improved health knowledge.[11]

In this study, among different sources of health information seeking, newspapers/magazines and Internet had stronger links with better SRH. Compared with saturation-level of diffusion of television and radio, information seeking from newspapers/magazines and Internet are more likely to be patterned by SES which may confound the results. To minimize the confounding effects, the associations were mutually adjusted for educational attainment and household income. Although mutual adjustment of different sources of health information in the same model may diminish some effects of these sources, the weak correlation among these sources suggested the associations might not be over-adjusted. Indeed, other studies have found higher levels of trust towards newspapers/magazines than television and radio,[12], [13] and trust towards health information is a driver for seeking behaviours.

Consistent with other studies,[10] SRH was socially patterned by educational attainment and household income among Hong Kong Chinese. More important, disparities in SRH was significantly and partially mediated by health information seeking inequalities with moderate proportions (7.9%–9.2%) of total mediation effect of SES on SRH. The finding was consistent with Western studies suggesting the contribution of health literacy and health information seeking on disparities in SRH among the elderly and cancer patients.[8], [14] Other studies have found that online health information seeking strengthened social support which may have led to the improvement in subjective health.[32] Online communication inequalities had also resulted in disparities in awareness and better knowledge on preventive measures such as human papillomavirus vaccination.[33]

Together with these findings, our results of mediating role of health information seeking on disparities in SRH supported the role of public communication on narrowing health inequalities.[34] Indeed, establishing information system to monitor health communication behaviours, such as The Health Information National Trends Survey (HINTS) in the United Sates,[35] will be the first step to provide useful evidence for informing the strategies to reduce health communication inequalities in Hong Kong and elsewhere. In particular, the rapid development of information and communication technologies (ICT) in mainland China provides unprecedented opportunities to improve individual and population health. Efforts to establish HINTS in mainland China have been initiated [36] and the findings may provide valuable evidence to improve health inequalities in this country with largest population in the world.

Our study had several limitations. The temporal sequence of health information seeking and SRH was uncertain due to the cross-sectional study design. The notion of reverse causality that better SRH led to more frequent health information seeking was not supported by the higher proportion of health information seeking among subject without chronic diseases, as reported in our previous study using same data.[16] In contrast, we found significantly lower frequency of health information seeking from television and Internet among subjects without chronic diseases. Other studies suggested having chronic diseases may trigger health information seeking,[6] although disease status had been adjusted in the modes of this study. Nevertheless, prospective studies are needed to confirm the findings. We only collected health information seeking from mass media and Internet, and did not include sources of medical professionals, family members and peers. This may underestimate overall health information seeking and bias the results in unknown directions. Although information seeking is an essential part of health communication, future studies should also measure other important components including attention to information, trust of information and level of self-efficacy on health information seeking. We are uncertain about the bias of non-response, incomplete and decreasing landline coverage on the findings. Future studies should consider other methods (e.g. dual sampling of landline and mobile telephone) to reduce such bias.

## Conclusions

Poor SRH was associated with lower socioeconomic status, and infrequent health information seeking from newspapers/magazines and Internet among Hong Kong Chinese. Disparities in SRH may be partially mediated by health information seeking from newspapers/magazines and Internet. The findings need to be confirmed using prospective data.
